# The Relation of Dentistry to Medicine

**Published:** 1881-10

**Authors:** J. B. McGregor


### THE RELATION OF DENTISTRY TO MEDICINE.

*Read before the Michigan Dental Association.*

BY J. B. McGREGOR.

The Michigan Dental Association, in its code of ethics, declares Dentistry to be a specialty in Medical Science; and as our profession advances the people more thoroughly recognize it as such. It is a subject which interests not only the dentist or physician but every human being requiring food, and teeth with which to masticate that food.

That diseased teeth are not conducive to general good health, nobody with any intelligence will for a moment argue. To educate people to the vital necessity of good teeth and the value of taking care of the same the assistance of all our friends is necessary. The physicians' relations being such that we court their assistance more than any other class. Though at present we stand somewhat apart, we hope ere long to go hand in hand, both striving in the same cause—the welfare of humanity. Dentistry of to-day has not that high standard of appreciation due it from the people. From the time of the first depositions for the formation till their end, the teeth are affected more or less by the general condition of the system, as well do they exert their influence upon the system. In the first stages of dentition, the irritation and inflammation produced by the coming tooth, will cause severe local suffering, besides producing at times disorders of sympathetic parts, ending it may be in fatal lesions of those parts.

Nervous derangements may occur or the inflammation may be such as to involve the salivary glands, the glands of the mouth or the absorbents and an endless amount of trouble may arise from this source. Dentition is also apt to aggravate any co-existing disease. A toothache is generally caused by some morbid state of the nerve or nerves, though it may be sympathetic with some morbid state of a distant organ, and in such cases the principal treatment should be directed to that part. Anything which deranges the digestive functions causes more or less indirectly the decay and degeneration of the teeth, through acid and corrosive eructations from the stomach or by so changing the condition of the fluids emptying into the mouth as to make them deteriorating instead of healthy useful agents. Our text-books teach us that to have proper digestion, the food should be first divided and comminuted so as to allow of the full action of the gastric, pancreatic juice and other digestive fluids; otherwise the full benefit is not derived. Assimilation will be imperfect and the body will show a lack of nourishment, thus making it more susceptible to disease. How is it with our friend whose teeth or surroundings or both are diseased? He has imperfect mastication because the parts being sore will not allow of the proper amount of pressure, and the grinding process is almost entirely dispensed with, the food is simply moistened with the saliva or some other fluid to allow the safe passage of the mouthful through the œsophagus. This is not complying with the laws of nature, and when we transgress in any one of nature's known laws other digressions will follow as will disastrous consequences. Thus it may be seen how the skill of our most eminent physicians will be baffled when the real cause of the disease may be traced back and its origin found in a mouthful of neglected teeth.

The same interest should be taken by medical men in the advancement of our profession as they take in the discovery of any new remedy which they expect in time to assist them in their work of prevention and eradication of disease. We are but branches of the same parent tree, each having a duty to perform, but together constituting the science of medicine. The physician will be called upon to treat diseases which have arisen from some derangement of the teeth but shows itself in some distant organ.

In treating such a disease it is essential to know the origin, that it may be successfully treated, and unless the physician has some knowledge of dentistry and can successfully treat the parent disease, the treatment of the offspring will often be long tedious and perhaps unsuccessful.

On the other hand we can not ignore the broader science of medicine, and practice with success, our favorite specialty.

Without a mutual interest and earnest labor for the advancement of the general science of medicine we will continue to have among us those practicing physicians and dentists who mistake the mere symptom for the disease, and of course the treatment based upon such an error will be useless if not actually hurtful.

